# Dystrophin deficiency reduces atherosclerotic plaque development in ApoE-null mice

**DOI:** 10.1038/srep13904

**Published:** 2015-09-08

**Authors:** Annelie Shami, Anki Knutsson, Pontus Dunér, Uwe Rauch, Eva Bengtsson, Christoffer Tengryd, Vignesh Murugesan, Madeleine Durbeej, Isabel Gonçalves, Jan Nilsson, Anna Hultgårdh-Nilsson

**Affiliations:** 1 Department of Experimental Medical Science, Lund University, Lund, Sweden; 2 Department of Clinical Sciences, Lund University, Malmoe, Sweden; 3 Department of Cardiology, Clinical Sciences, Lund University, Malmoe, Sweden

## Abstract

Dystrophin of the dystrophin-glycoprotein complex connects the actin cytoskeleton to basement membranes and loss of dystrophin results in Duchenne muscular dystrophy. We have previously shown injury-induced neointima formation of the carotid artery in mice with the *mdx* mutation (causing dystrophin deficiency) to be increased. To investigate the role of dystrophin in intimal recruitment of smooth muscle cells (SMCs) that maintains plaque stability in atherosclerosis we applied a shear stress-modifying cast around the carotid artery of apolipoprotein E (ApoE)-null mice with and without the *mdx* mutation. The cast induces formation of atherosclerotic plaques of inflammatory and SMC-rich/fibrous phenotypes in regions of low and oscillatory shear stress, respectively. Unexpectedly, presence of the *mdx* mutation markedly reduced the development of the inflammatory low shear stress plaques. Further characterization of the low shear stress plaques in ApoE-null *mdx* mice demonstrated reduced infiltration of CD3^+^ T cells, less laminin and a higher SMC content. ApoE-null *mdx* mice were also found to have a reduced fraction of CD3^+^ T cells in the spleen and lower levels of cytokines and monocytes in the circulation. The present study is the first to demonstrate a role for dystrophin in atherosclerosis and unexpectedly shows that this primarily involves immune cells.

Atherosclerosis is the most important cause of acute cardiovascular events including myocardial infarction and stroke^1^. An important step in the development of atherosclerotic plaques is the binding of lipids to proteoglycans of the extracellular matrix (ECM)^2^, and the subsequent modification/oxidation and uptake of these lipids by intimal macrophages^3^. Lipid-filled macrophages accumulate within the lesion as foam cells^4^, which, together with the endothelial cells, produce cytokines and growth factors that activate vascular smooth muscle cells (SMCs) in the media of the artery wall^5^,^6^.

In the vessel wall endothelial cells reside on and SMCs are surrounded by a basement membrane, that, in addition to providing structural support, functions as a growth factor reservoir, directs tissue organization and presents binding sites for cell adhesion molecules and ligands for cell receptors, as well as affects cell signalling, differentiation, proliferation and migration^7^. The basement membrane consists mainly of the ECM components laminin, collagen type IV (a non-fibrillar collagen), perlecan and nidogen^8^.

Dystrophin, an intracellular protein located at the cytoplasmic face of the plasma membrane in both SMCs and skeletal muscle cells^9^,^10^,^11^,^12^, connects the actin cytoskeleton to perlecan and laminin α2-chains in the basement membrane^13^. The connection is made through association with the dystrophin-glycoprotein complex (DGC), comprised of transmembrane and cytosolic proteins. In addition to dystrophin, it includes α- and β-dystroglycans, α-, β-, γ- and δ-sarcoglycans, sarcospan and the syntrophins^14^,^15^,^16^,^17^. The DGC in skeletal muscle is thought to protect muscle fibres from damage caused by mechanical forces during muscle contraction^13^,^18^,^19^.

Loss of dystrophin expression leads to Duchenne muscular dystrophy (DMD; an X-linked recessive muscle disorder), which is the most common form of muscular dystrophy affecting one in 3500 male births^20^,^21^. Muscle wasting is progressive and the life span of afflicted patients is shortened to 20–30 years^22^. Similarly, the *mdx* (X-chromosome-linked muscular dystrophy) mouse^23^, that lacks full-length dystrophin due to a point mutation^24^,^25^, also develops muscular dystrophy, though in a much milder form compared to DMD patients^26^.

We have previously found evidence for a role of dystrophin in activation of the repair processes carried out by SMC in injured arteries^27^. These studies showed that SMCs down-regulate dystrophin as they de-differentiate from contractile to synthetic phenotype and that *mdx* mice are characterized by increased neointima formation in response to collar injury of the carotid artery. Based on this work along with several previous reports of an altered vasculature in the *mdx* mouse^28^,^29^,^30^ we hypothesize that dystrophin may play a role in atherosclerosis by controlling the recruitment of medial SMCs into the lesions, a process of critical importance for maintaining plaque stability. To test this possibility that dystrophin-deficiency would lead to the formation of larger and more SMC-rich plaques we generated *mdx* mice on an Apolipoprotein E (ApoE)-null background.

## Materials and Methods

Materials and Methods may be found in more detail in the online data supplement.

### Animals and *In Vivo* Alteration of Shear Stress

ApoE-null mice (a gift from Prof. Stefan Jovinge, Lund University, Sweden) were crossed with mdx mice (C57/10ScSn-mdx/J from Jackson Laboratory). For the current experiments ApoE-null mdx (ApoE/mdx) mice were used with ApoE-null mice as controls. Periadventitial cast placement to produce standardized changes in shear stress was performed as described previously by Cheng *et al.*^31^. Briefly, casts were placed around the right carotid artery of female 18-week-old mice. Mice were kept on a Western diet (R368: 0.15% cholesterol, 21% fat from Lantmännen, Sweden) starting two weeks before surgery until sacrifice at 30 weeks of age. All experiments involving mice were approved by the Malmö/Lund Ethical Committee for Animal Research (Sweden) and were carried out in accordance with the approved guidelines.

### Histology, immunohistochemistry and immunofluorescence

Flat preparations of aortas were stained with Oil Red O.

For immunofluorescence, antibodies against laminin (α1/β1/γ1 chains, L9393, Sigma-Aldrich, Stockholm, Sweden) and Caspase-3 (ab4051, Abcam, Cambridge, UK) were used with a was Cy3-conjugated secondary antibody (goat anti rabbit Ig, Jackson ImmunoResearch Laboratories Inc. Baltimore Pike, PA, USA).

Sections of mouse carotid artery plaque were stained using primary antibodies against smooth muscle α-actin (α-SMA; clone 14A, Sigma-Aldrich), CD31 (clone SZ31, Dianova GmbH, Hamburg, Germany), mac2 (Cedarlane; Burlington, ON, Canada), CD3(ab16044-100, Abcam) and smooth muscle myosin heavy chain 11 (ab125884, Abcam). Sections were counterstained with Mayer’s hematoxylin (Histolab, Västra Frölunda, Sweden).

### Analysis of plasma cytokines and cholesterol

The colorimetric assay Infinity Total Cholesterol (Thermo Scientific, Liverpool, U.K.) was used to quantify total plasma cholesterol and the Bio-Plex Pro Mouse Cytokine Assay (BIO-RAD, Hercules, CA, USA) was used to quantify plasma cytokine concentrations of interleukin (IL)-2, IL-4, IL-5, IL-6, IL-10, IL-12p70, IL-13, IL-17A, interferon-γ (IFNγ) and tumour necrosis factor-α (TNF-α). Both analyses were performed according to instructions from the manufacturer.

### Flow cytometry

Splenocytes and blood leukocytes from ApoE-null and ApoE/*mdx*- mice were stained with flourochrome-conjugated antibodies and were captured and analysed with a CyAn ADP flow cytometer (Beckman Coulter, High Wycombe, UK) using Summit software (Dako, Fort Collins, Colorado, USA). Erythrocytes were removed using red blood cell lysing buffer (Sigma, St. Louis, MO, USA). Fc receptors were blocked before extra- and intracellular staining (FcR; CD16/32). The following antibodies were used; CD3-PE/Cy7, CD4-PB, IL-5-PE, IFNγ-PE, IL-17-APC, Ly6c-PE/Cy7 and CD115-APC (Biolegend, San Diego, CA).

### Statistical methods

Image analysis was performed using BioPix iQ software (BioPixAB, Gothenburg, Sweden). Positive immunoreactivity was quantified and expressed as percentage positively stained lesion area (relative to the total lesion size). Immunofluorescence was scored blindly on a scale from 1 to 5 according to the extent of positive staining in the lesion (with 5 representing the most extensive immunofluorescence). Lesion size of carotid artery plaques is expressed as intima/media ratio and area (μm^2^) and is the mean value of four sections 15 μm apart collected where each lesion was at its largest.

Samples were not normally distributed and Mann-Whitney U test (two-tailed) was performed using GraphPad Prism version 6.05 for Windows, GraphPad Software, San Diego, California, USA, www.graphpad.com). P-values < 0.05 were considered statistically significant. Values are presented as median with interquartile range (25th percentile to 75th percentile; IQR) with error bars representing IQR in graphs. Sample size is expressed as n.

## Results

In order to investigate how the connection between cells and basement membranes influences atherogenesis, atherosclerotic lesions were induced in Apolipoprotein E (ApoE)-null mice and ApoE-null mice with the *mdx* mutation (referred to as ApoE and ApoE/*mdx* mice throughout this report) through a shear stress-modifying periadventitial cast placed around the right carotid artery^31^. The cast is thus placed around non-curved, non-branched region of the carotid artery where plaques do not naturally form—even in ApoE mice given a Western diet (as in the present study). Due to the tapered shape of the cast, a region of low shear stress induced plaques of a more inflammatory phenotype (i.e. containing a higher ratio of macrophages) proximal to the cast, and the region of oscillatory shear stress induced plaques of a more fibrous phenotype (i.e. containing a higher ratio of collagenous ECM) distal to the cast (Supplementary Fig. S1). The endothelium remained intact in both types of lesions, as shown by CD31 immunoreactivity (Supplementary Fig. S1). In the region of high shear stress inside the cast no plaques were formed. There was no difference in plasma levels of cholesterol or triglycerides in ApoE compared to ApoE/*mdx* mice (Supplementary Fig. S2).

### Lack of dystrophin reduces plaque formation in the low-shear stress region and in the aorta

**Figure 1:**
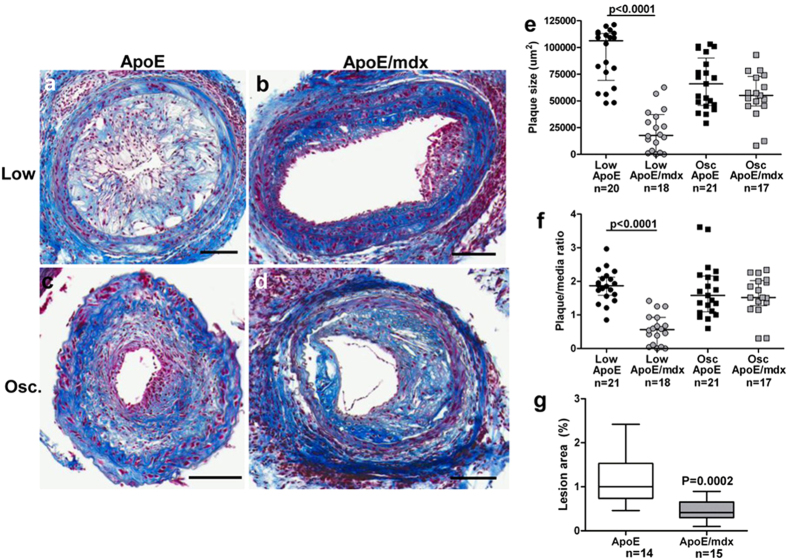
Reduced atherosclerotic burden in ApoE/*mdx* mice. Representative sections of Masson’s trichrome stain from low shear stress (**a**–**b**) and oscillatory shear stress (**c**–**d**) plaques in ApoE- and ApoE/*mdx* mice. Plaque size represented by plaque/media ratio (**e**) and total plaque size (**f**). Quantification of Oil Red O-stained flat preparations of aortas from ApoE and ApoE/*mdx* mice (**g**). Scale bars represent 100 μm and Mann-Whitney U test was used.

In the ApoE/*mdx* mouse, carotid artery plaques developing in the low shear stress region were smaller than the plaques formed in the same region in ApoE mice (P < 0.0001, Fig. 1a–f). Plaque sizes in the oscillatory shear stress region were similar in ApoE and ApoE/*mdx* mice. Development of atherosclerosis in the aorta, as assessed by *en face* Oil Red O staining, was reduced by almost 60% in ApoE/*mdx* mice as compared to ApoE mice (Fig. 1g and Supplementary Fig. S3).

### Dystrophin deficiency increases the SMC content of low shear but not of oscillatory shear stress plaques

**Figure 2:**
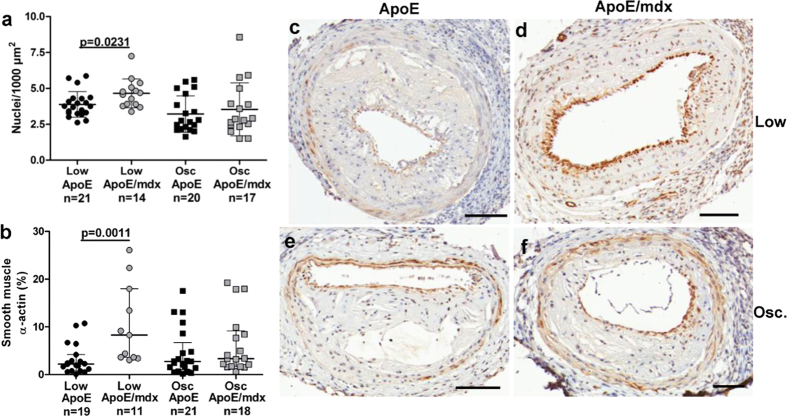
Increases in cell density and smooth muscle cells. Increased cell density (**a**) and smooth muscle cell content (**b**)—the latter as measured by the relative content of smooth muscle α-actin (α-SMA)—in low shear stress plaques from ApoE/*mdx* mice compared to ApoE mice. Representative α-SMA stained sections are shown in (**c**–**f**). Scale bars represents 100 μm and Mann-Whitney U test was used.

Along with smaller size, increased cell density was found in low shear stress plaques from ApoE/*mdx* compared to low shear stress plaques from ApoE mice (P = 0.0231, Fig. 2a). Smooth muscle α-actin (α-SMA) immunoreactivity—primarily found in the fibrous cap—was increased in low shear stress-plaques from ApoE/*mdx* mice compared to plaques from the same region in ApoE mice (2.2 (0.8–4.2)% versus 8.3 (3.6–18.0)%, P = 0.0011; Fig. 2b–f). These plaques also contained increased amounts of smooth muscle myosin heavy chain (p = 0.0048, Supplementary Fig. S4), another SMC marker associated with a differentiated phenotype^32^. SMC content as assessed by both markers was similar in oscillatory shear stress-plaques of both genotypes. There was no difference in cell proliferation (Ki67), apoptosis (caspase-3) or in the ratios of macrophage (mac-2) or endothelial cell (CD31) immunoreactivity in either type of plaque (Supplementary Fig. S1, S5 and S6). Consequently, the increase in SMCs appears to account for the increased cell density found in low shear stress-plaques from ApoE/*mdx* mice.

### Lack of dystrophin affects laminin expression in the plaque/lumen interface

**Figure 3:**
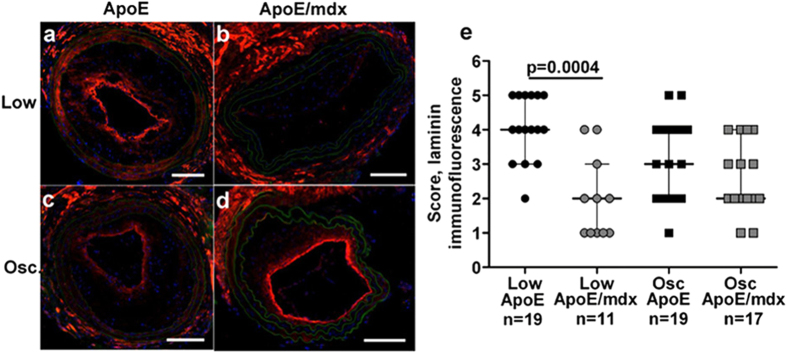
Decreased immunoreactivity for laminin in low shear stress plaques in ApoE/*mdx* compared to ApoE mice. Representative images showing laminin immunoreactivity in low shear stress plaques are found in (**a**,**b**), and oscillatory shear stress plaques in (**c**,**d**). Plaques from ApoE mice are found in (**a**,**c**), and plaques from ApoE/*mdx* mice in (**b**,**d**). Quantification are found in e. Nuclei are stained blue with DAPI and autofluorescence is shown in green. Scale bars represent 100 μm and Mann-Whitney U test was used.

In the smaller low shear stress plaques of ApoE/*mdx* mice we also found the laminin expression to be altered. Utilizing an antibody against laminin chains α1, β1 and γ1, thereby detecting the vast majority of the laminin isoforms, we found that low, but not oscillatory, shear stress carotid plaques from ApoE/*mdx* mice contained less laminin compared to plaques from ApoE mice (p = 0.0004, Fig. 3). Laminin-positive immunoreactivity was typically found in the media and in the plaque region facing the vessel lumen.

### Lack of dystrophin decreases accumulation of CD3^+^ T cells in low shear stress-plaques

**Figure 4:**
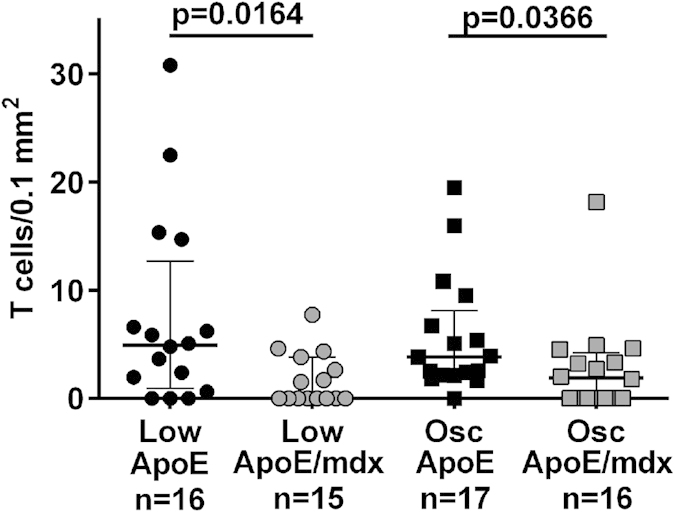
Decrease in T cell plaque content. The number of T cells relative to plaque size was decreased in low shear stress plaque from ApoE/*mdx* compared to ApoE mice (as determined through Mann-Whitney U test).

To determine if the reduced development of atherosclerosis in ApoE/*mdx* mice involved changes in vascular inflammation we analysed the macrophage and T cell accumulation in plaques from the low and oscillatory shear stress region of the carotid artery. Plaques from ApoE/*mdx* demonstrated a marked reduction in CD3^+^ T cell numbers in both low shear stress (0 (0–3.8) versus 5.0 (1.0–12.7) cells/0.1 mm^2^, p = 0.02 and oscillatory shear stress lesions (1.9 (1.0–4.2) versus 3.8 (2.2–8.1) cells/0.1 mm^2^, p = 0.04) (Fig. 4 and Supplementary Fig. S7), while there was no difference in macrophage immunoreactivity (Supplementary Fig. S6).

### Dystrophin deficiency affects the immune cell composition in the spleen and circulation

**Figure 5:**
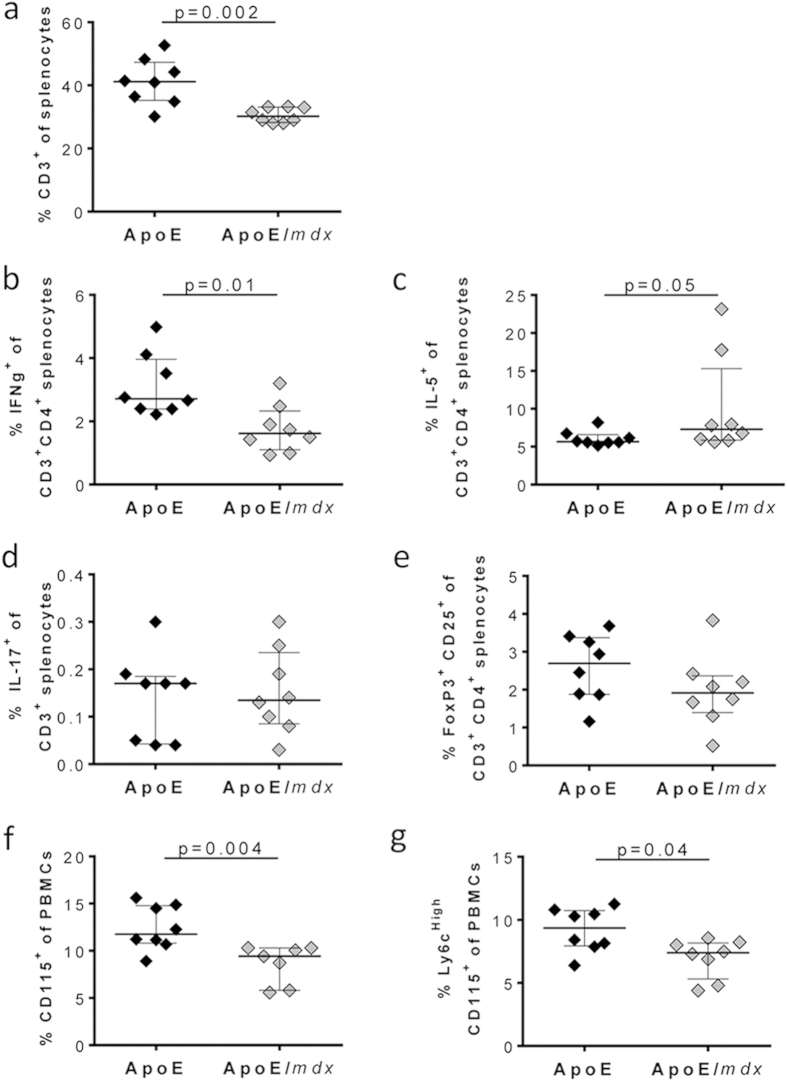
Lack of dystrophin in high fat diet fed apoE mice results in decreased T cells and monocytes. Quantification of frequencies of CD3^+^ splenocytes (**a**), IFNγ^+^ (**b**), IL5^+^ (**c**), IL-17^+^ (**d**) and FoxP3^+^ CD25^+^ (**e**) among T cells, and CD115^+^ (**f**) and Ly6C^High^ CD115^+^ (**g**) among PBMCs with comparisons made through the Mann-Whitney U test.

To determine if the reduced accumulation of CD3^+^ T cells in atherosclerotic lesions of ApoE/*mdx* mice reflected a general effect of dystrophin deficiency on the immune system we analysed T cell and monocyte populations in the spleen and in the circulation. ApoE/*mdx* mice were found to have a lower fraction of CD3^+^ cells in spleen (Fig. 5a). The fraction of both CD3^+^ CD4^+^ and CD3^+^ CD4^−^ T cells were reduced in ApoE/*mdx* as compared with in ApoE mice (9.7 (8.9–10.5)% versus 16.2 (11.9–18.4)%, p = 0.0006) and 21.0 (19.0–23.2)% versus 25.1 (22.5–29.5) %, p = 0.02; respectively). Further characterization of the CD4^+^ T cells revealed decreased IFNγ^+^ T cells, but increased IL5^+^ T cells in ApoE/*mdx* mice, whereas Tregs (CD25^+^ FoxP3^+^) or IL17^+^ CD3^+^ cells did not differ between ApoE/*mdx* mice or ApoE mice (Fig. 5b–e). In addition, ApoE/*mdx* mice displayed a lower fraction of monocytes in blood, as well as decreased inflammatory Ly6c^High^ monocytes (Fig. 5f–g).

To investigate if the differences in immune cells were due to the Western diet, we analysed immune cells in chow fed mice. The fraction of CD3^+^ splenocytes was decreased also in chow fed ApoE/*mdx* mice (Supplementary Fig. S8). Furthermore, both CD3^+^ CD4^+^ (13.9% (12.4–15.8)% versus 19.2% (16.9–20.9)%, p = 0.001) and CD3^+^ CD4^−^ (17.7% (16.5–17.9)% versus 19.8% (19.0–21.5)%, p = 0.007) fractions were decreased in ApoE/*mdx*. The fractions of monocytes or inflammatory Ly6C^high^ monocytes in blood did not differ between chow fed ApoE/*mdx* and ApoE mice (Supplementary Fig. S8).

### Lack of dystrophin results in an altered cytokine profile in plasma

As the development of both carotid and aortic plaques was affected in the ApoE/*mdx* mouse, we investigated whether any systemic effects on inflammatory response existed in this mouse. Indeed, the cytokines interleukin (IL)-2, −4, −6, −10, −12p70, −13, −17 and IFNγ were all down-regulated in plasma from ApoE/*mdx* mice compared to the ApoE mice (Table 1). The cytokine profile was similarly affected in mice that had been given regular chow with a down-regulation of IL-2, −4, −6, −13 and TNFα ApoE/*mdx* mice, demonstrating that this effect was not dependent on the Western diet (Supplementary Table S1).

## Discussion

Dystrophin is primarily recognized for its role in connecting muscle cells to the surrounding ECM. We have previously shown that vascular SMCs down-regulate dystrophin as they modulate from expressing a contractile to synthetic phenotype, a critical phenomenon in vascular repair responses as well as in the formation of atherosclerotic plaques. Lack of dystrophin was also found to be associated with enhanced neointima formation in response to carotid injury in mice. Against this background we postulated that ApoE/*mdx* mice would develop larger, more stable and SMC-rich atherosclerotic lesions. However, although the inflammatory low shear stress lesions indeed were found to contain more SMCs there was no difference in either size or SMC content in the more fibrous oscillatory shear stress lesions. Moreover, there was an unexpected reduction of plaque development both at the low shear stress site and in the aorta. This was accompanied by a marked reduction of T cell infiltration in the plaques as well as a more general suppression of immunity with reduced T cell levels in the spleen, lower plasma cytokines and less circulating monocytes including the inflammatory Ly6C^high^ type. Taken together these observations (summarized in Suppl. Fig. S9) support the notion that dystrophin is involved in controlling the recruitment of SMC into inflammatory atherosclerotic lesions but unexpectedly also the dystrophin play an even more important role in atherosclerosis by controlling the immune responses that contributes to plaque development. Notably, whereas atherosclerotic plaques in ApoE/*mdx* mice contained markedly less T cells there was no difference in the macrophage content.

The effect of dystrophin deficiency on the immune system is not well characterized. The muscular dystrophy in Duchenne patients and *mdx* mice is associated with local inflammatory responses believed to contribute to myofiber death^33^,^34^. However, the inflammatory process in Duchenne differs from that of most other autoimmune and infectious myopathies in that fewer T cells accumulate in affected tissues^35^,^36^. The reason for the relative lack of T cell involvement in the inflammatory response to Duchenne muscular dystrophy remains to be fully elucidated, but Cascabulho and co-workers have shown that T cells from *mdx* mice are characterized by reduced endothelial homing due to shedding of CD62 ligand. These observations are well in line with the reduced accumulation of T cells found in atherosclerotic plaques of ApoE/*mdx* mice in our study. Additionally, we observed a relative decrease in the spleen T cell content as well as reduced levels of T cell-derived cytokines in plasma in ApoE/*mdx* mice suggesting that dystrophin deficiency may influence the development of the immune system. T cells are known to play an important role in the development of atherosclerosis and it is reasonable to assume that the reduced development of atherosclerosis in ApoE/*mdx* mice at least in part is explained by a lower T cell recruitment into lesion-prone arterial sites. Among CD4^+^ T cells, T_H_1 cells are considered pro-atherogenic while a T_H_2 response appears to have atheroprotective qualities^37^ characterization of CD4^+^ T cells isolated from the ApoE/*mdx* spleen revealed a T cell response that appeared to favour the more atheroprotectiveT_H_2 rather than aT_H_1 response; another possible factor in the development of smaller low shear stress plaques in ApoE/*mdx* mice. A molecular mechanism explaining the reduced fraction of CD3^+^ T cells in the spleen can refer to modifications of the basement membrane surrounding the dystrophin-defective SMCs and endothelial cells. The T cells enter the white pulp of the spleen through open arterioles, which contains one to two layers of SMCs and one hypothesis would be that the altered basement membrane, including less laminin, would make adhesion and migration of the T cells less effective. A similar phenomenon may also explain the reduced infiltration of CD3^+^ T cells in the inflammatory low shear stress lesions.

Observations of reduced circulating levels of IL-6 and pro-inflammatory Ly6C^high^ monocytes in ApoE/*mdx* mice also suggested a general dampening of inflammatory activation as a result of dystrophin deficiency. These findings are in contrast to a previous study^35^ that found expression of several cytokines, including IL-2, −4 and TNFα, to be elevated in muscle and spleen of *mdx* compared to wild type mice. However, the possible discrepancy between these results may be explained by our use of ApoE mice that have higher levels of circulating low density lipoprotein (LDL)—in this study, also exacerbated by fatty diet—that likely serves as inflammatory stimuli, possibly affecting baseline cytokine profiles. It is thus interesting to note that the ApoE/*mdx* mouse, while under the influence of two possible inflammatory stressors—potentially inflamed skeletal muscle, as reported in the study by Vetrone *et al.*^35^, and circulating LDL—has lower plasma cytokine levels. Indeed, together with the decreased growth of low shear stress plaques in ApoE/*mdx* mice—plaques with an inflammatory phenotype—one can hypothesize a muted effect on the response of immune cells in these mice, involving a decreased response to inflammatory stimulus or altered release of inflammatory stimulus, such as cytokines, or a combination of the two.

The original hypothesis with our study was that ApoE/*mdx* mice would develop larger and more SMC-rich atherosclerotic plaques. With the exception of an increased content of SMC in the inflammatory low shear stress plaques this turned out not to be the case. We have previously shown that *mdx* mice are characterized by increased neointima formation in response to collar injury of the carotid artery. This neointima is formed by SMC recruited from the media and the process resembles in many ways the fibrous plaques that develop in the oscillatory shear stress region of the ApoE/*mdx* mice on high fat diet. The lack of effect of dystrophin deficiency on the formation of these plaques remains to be elucidated but may be due to the increased complexity of the atherosclerotic disease process as compared with the repair responses to mechanical injury. While the mechanically induced lesions in our first study^27^ consisted almost solely of SMCs and ECM, the atherosclerotic lesions in the present study develop due to modifications in fluid shear stress rather than mechanical stress. In addition, this model includes the atherosclerosis-prone ApoE-null mouse on a fatty diet. In this mouse, lipids as well as inflammatory cells and necrotic areas are found in lesions induced by shear stress—therefore the resulting lesions more closely resemble the human atherosclerotic plaque. Thus, the additional influence of lipids and inflammation on lesion development may explain why lesion size was affected differently by the *mdx* mutation in the two experimental models used. In favour of this hypothesis is also the finding that there was no difference in plaque size between genotypes in the oscillatory shear stress region. These plaques represent an intermediate plaque phenotype; while they do contain inflammatory cells, the relative macrophage content is significantly decreased and the collagen content significantly increased in comparison to low shear stress plaques^38^.

The increase in SMCs in the inflammatory low shear stress plaques may be explained by an increased migration by these cells. The SMC in the artery wall is highly dependent on an intact basement to remain in a differentiated phenotype. Once the composition of the basement membrane is being altered/degraded the SMC becomes activated with capacity to both migration and proliferation. It has been demonstrated that especially the laminin content is of importance^39^,^40^. The non-functional dystrophin in the mdx mouse results in altered composition in the basement membrane and a subsequent disrupted connection between the SMC, the basement membrane and the ECM^18^. It is likely that this environment favors a transition to a more de-differentiated phenotype with increased migration and proliferation. Indeed, increased migration has been found in human mesenchymal tumor cells with DMD deletions which inactivate the larger dystrophin isoform^41^.

Dystrophin is expressed in vascular SMCs as well as in endothelial cells^12^,^28^,^42^,^43^. Expression has been reported in large arteries as well as veins with contractile properties, and dystrophin is thus suggested to have an active part in the mechanical resistance of the muscle membrane^44^. While shear stress itself is not altered in *mdx* mice, Loufrani *et al.*^28^ found that the ability of endothelial cells to produce the vasorelaxant nitric oxide (NO) in response to shear stress was decreased. The same group later also observed inability of *mdx* arteries to adapt to chronic changes in blood flow with diameter and wall thickness increase or endothelial NO synthase expression and NO-dependent dilation^29^. Altered biomechanical properties in *mdx* carotid arteries were also reported by Dye *et al.*^30^, in that these arteries exhibited greater circumferential and axial stiffness. Dystrophin tends to be found in terminally differentiated cells^20^, and, with the exception of the cells lining the lumen and making up the fibrous cap, SMCs found in atherosclerotic plaques appear to exist in a more dedifferentiated state as activation and phenotypic modulation is required in order to leave the tunica media. As a result, dystrophin expression in SMCs in the deep plaque tissue is likely down-regulated in the same way as for example α-SMA and smooth muscle myosin heavy chain. We have previously analysed expression of dystrophin, along with β-dystroglycan and β-sarcoglycan, in primary aortic murine SMCs *in vitro*^27^, and found mRNA for all three proteins to be heavily reduced as the SMC phenotype switched from a contractile to a more synthetic state. This may indicate a lower degree of dependence on ECM interaction via the DGC as dedifferentiated SMCs change their expression of surface receptors^45^, such as integrins, to be able to bind to additional types of collagen and laminin chains.

In conclusion, with this study we present for the first time a role for dystrophin and the DGC in atherosclerotic plaque development. The results demonstrate that absence of dystrophin in an ApoE-null background results in attenuated atherosclerotic plaque development, accompanied by an increased proportion of differentiated SMCs and decreased proportion of T cells in plaques of an inflammatory phenotype. One potential underlying mechanism is altered involvement by the immune system and our study provides novel insight of a potential role for dystrophin in immunity.

## Additional Information

**How to cite this article**: Shami, A. *et al.* Dystrophin deficiency reduces atherosclerotic plaque development in ApoE-null mice. *Sci. Rep.* **5**, 13904; doi: 10.1038/srep13904 (2015).

## Supplementary Material

Supplementary Information

## Figures and Tables

**Table 1:** Cytokine levels in plasma from ApoE and ApoE/*mdx* mice on a high-fat diet (n = 28 for both genotypes).

**Cytokines**	**Plasma levels (pg/ml)**		**P values**
	**ApoE**	**ApoE/*mdx***	
IL-2	30.1 (22–50.5)	19.41 (10.1–27.8)	0.0005
IL-4	14.6 (11.3–17.6)	9.9 (7.8–11.9)	<0.0001
IL-5	46.4 (30.7–55.5)	32.8 (14.2–43.3)	NS
IL-6	25.2 (17.2–40.1)	20.0 (11.9–28.1)	0.0456
IL-10	155.6 (90.1–205.1)	91.1 (47.2–164.58)	0.0414
IL-12p70	550.2 (467.7–765.4)	409.5 (278.7–564.2)	0.0150
IL-13	1431 (733.6–1920)	930 (155.0–1269)	0.0101
IL-17	60.5 (38.3–78.9)	43.1 (29.3–59.3)	NS
IFNγ	30.3 (14.2–39.7)	17.7 (5.2–30.3)	0.0303
TNFα	1384 (778.3–1936)	1532 (839.2–1532)	NS

The Mann-Whitney U test was used and values are shown as median with interquartile range.
