# Supplementary material for: Dystrophin deficiency reduces atherosclerotic plaque development in ApoE-null mice
Source: Sci Rep. 2015 Sep 8;5:13904. doi: 10.1038/srep13904 (PMC4561962; doi:10.1038/srep13904)
Supplement: Supplementary Information [file srep13904-s1.pdf]

Dystrophin deficiency reduces atherosclerotic plaque development in ApoE-null mice

Annelie Shami[1], Anki Knutsson[1], Pontus Dunér[2], Uwe Rauch[1], Eva Bengtsson[2], Christoffer Tengryd[2], Vignesh Murugesan[1], Madeleine Durbeej[1], Jan Nilsson[2], Isabel Gonçalves[2, 3], Anna Hultgårdh-Nilsson[1]

1. Department of Experimental Medical Science, Lund University, Lund, Sweden
2. Department of Clinical Sciences, Lund University, Malmoe, Sweden
3. Department of Cardiology, Clinical Sciences, Lund University, Malmoe, Sweden

SUPPLEMENTARY INFORMATION

Supplementary methods

Animals and In Vivo Alteration of Shear Stress
ApoE-null mice (a gift from Prof. Stefan Jovinge, Lund University, Sweden) were crossed with mdx mice (C57/10ScSn-*mdx*/J from Jackson Laboratory). For the current experiments ApoE-null mdx (ApoE/*mdx*) mice were used with ApoE-null mice as controls. Periadventitial cast placement to produce standardized changes in shear stress was performed as described previously by Cheng et al.[1] Briefly, casts were placed around the right carotid artery of female 18-week-old mice anaesthetized with isoflurane carried by oxygen (initiated at 4%, maintained at 2-3% during surgery). Mice were given buprenorhine (0.1 mg/kg) subcutaneously once before surgery and once five to seven hours post-surgery. Mice were kept on a Western diet (R368: 0.15% cholesterol, 21% fat from Lantmännen, Sweden) starting two weeks before surgery until sacrifice at 30 weeks of age (achieved by administration of an overdose of ketamine, xylazin and acepromazine). All experiments involving mice were approved by the Malmö/Lund Ethical Committee for Animal Research (Sweden) and was carried out in accordance with the approved guidelines.

Preparation of mouse tissue
Blood was collected from the right chamber of the heart, and spleens were removed. Mice were then either perfused with HistoChoice tissue fixative (Amresco, Solon, OH, USA) before carotid arteries, aortas and skeletal muscle were removed and fixed for several days with HistoChoice; or perfused with PBS before carotid arteries were removed and embedded in optimal cutting medium (Sakura Finetek, Japan), frozen directly and sectioned (7µm). Tissue fixed with HistoChoice was rinsed in PBS, dehydrated, embedded in paraffin and sectioned (5 µm). Descending aortas were rinsed in PBS, cleaned and mounted en face with ovalbumin/glycerin (Sigma-Aldrich, Stockholm, Sweden).

Histology, immunohistochemistry and immunofluorescence
Mouse carotid artery sections were stained with Masson's trichrome stain ("Accustain trichrome (Masson)", Sigma-Aldrich) following the manufacturer's instructions. Flat preparations of aortas were stained with 0.3% Oil Red O for 50 minutes and mounted with Mountquick (Daido Sangyo Co. LTD, Tokyo, Japan).

For immunofluorescence, sections were fixed in methanol, and antibodies against laminin (α1/β1/γ1 chains, L9393, Sigma-Aldrich, Stockholm, Sweden) and Caspase-3 (ab4051, Abcam, Cambridge, UK) were used for carotid artery-sections that were paraffin-embedded and frozen, respectively. The secondary antibody was Cy3-conjugated (goat anti rabbit Ig, Jackson ImmunoResearch Laboratories Inc. Baltimore Pike, PA, USA) and slides were mounted with Fluoromount (Sigma-Aldrich).

Sections of mouse carotid artery plaque embedded in paraffin were stained using antibodies against smooth muscle α-actin (α-SMA; clone 14A, Sigma-Aldrich), CD31 (clone SZ31, Dianova GmbH, Hamburg, Germany) and mac2 (Cedarlane; Burlington, ON, Canada). Sections were deparaffinized and rehydrated in xylene and a graded series of alcohols before heat-induced antigen epitope retrieval was performed (pH 6.0, 12 minutes). Frozen sections of mouse carotid artery plaques were fixed with acetone, permeabilised with 0.1% Triton X-100 and stained using antibodies against CD3 and smooth muscle myosin heavy chain 11 (ab16044-100 and ab125884, respectively – both from Abcam, Cambridge, UK). For stainings using the α-SMA antibody, the MOM-kit was used, followed by the Vectastain Elite Kit and for other antibodies the ImmPRESS Anti-Rabbit or Rat (mouse-adsorbed) Ig (peroxidase) Polymer Detection Kits were used according to the manufacturer's instructions (all from Vector Labs, Burlingame, CA, USA). Immunoreactivity was visualized with 3,3'-diaminobenzidine (DAB) and sections were counterstained with Mayer's hematoxylin (Histolab, Västra Frölunda, Sweden).

Immunohistochemically stained sections were scanned and digitalized using an Aperio ScanScope digital slide scanner (Scanscope Console v8.2.0.1263, Aperio Technologies, Inc., Vista, California, USA) and images of immunofluorescent stains were taken using a Zeiss Axiophot 2 with a Hamamatsu C4742-95 camera, an X-Cite series 120Q lamp (Lumen Dynamics) and Openlab 5 software (Improvision).

Splenocyte isolation and cell culture

Spleens taken from ApoE-null and ApoE/*mdx* mice that had not undergone surgery, but had been kept on a Western diet or regular chow for 14 weeks, were used for cytokine production and flow cytometry analyses. Briefly, single cell suspensions were prepared by pressing spleens through a 70-µm cell strainer (BD Falcon, Franklin Lakes, NJ). Erythrocytes were removed using red blood cell lysing buffer (Sigma-Aldrich). Cells were cultured in culture medium containing 10% heat-inactivated FCS, 1 mmol/L sodium pyruvate, 10 mmol/L Hepes, 50 U of penicillin, 50 µg/mL streptomycin, 0.05 mmol/L β-mercaptoethanol, and 2 mmol/L L-glutamine (RPMI 1640, GIBCO, Paisley, UK) in 48-well bottom plates. For the T-cell assessment by flow cytometry, 5×105 cells/well were cultured with or without 20 ng/mL PMA, 1 ug/ml Ionomycin and 5 ug/ml Brefeldin A (Sigma-Aldrich) for 4 (IL-5 and IFNγ expression) or 24 (IL-17A expression) hours.

Supplementary figures

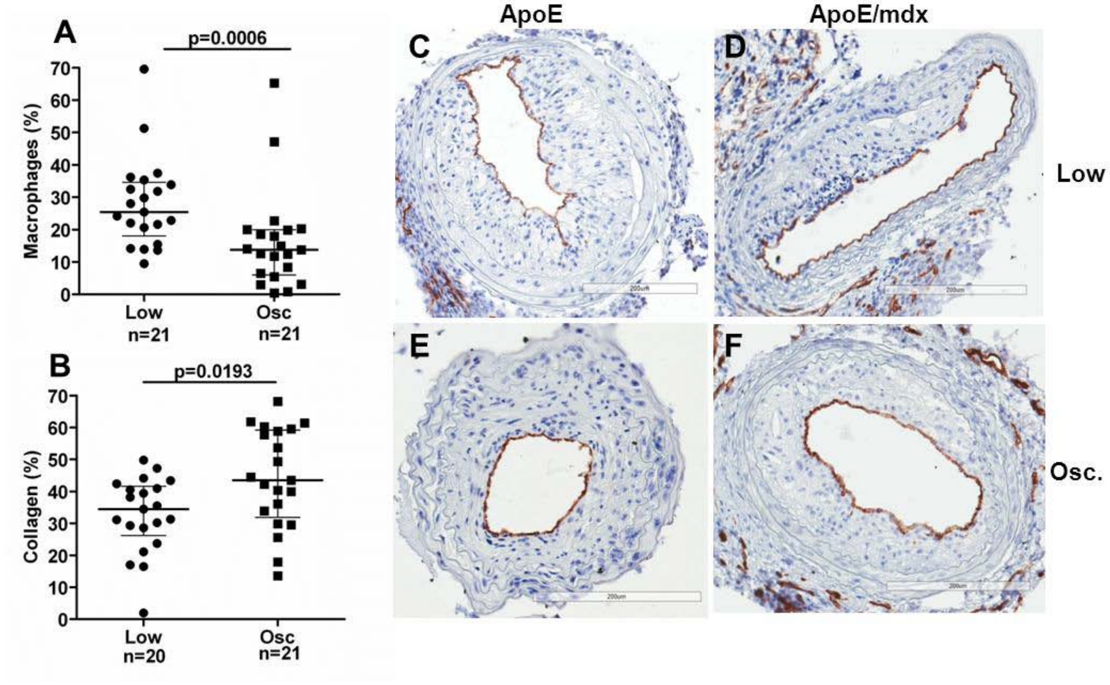

Supplementary figure S1: Characterisation of plaques induced by the shear stress-modifying periadventitial cast. Decreased macrophage content (a) and increased collagen content (b) in low versus oscillatory shear stress lesions from ApoE-null mice. Endothelium remains intact in plaques induced by the shear stress-modifying cast; representative micrographs show CD31 immunoreactivity in low (c-d) and oscillatory (e-f) shear stress lesions of both genotypes (ApoE in c and e, ApoE/*mdx* in d and f). Scale bars represent 200 µm and and Mann-Whitney U test was used.

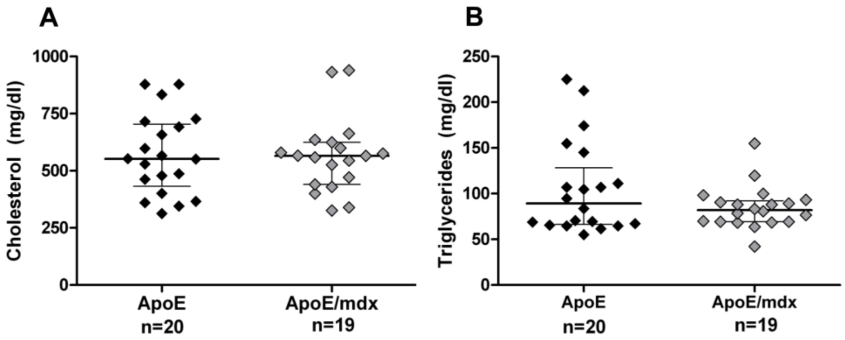

Supplementary figure S2: Plasma cholesterol and triglycerides. Levels of cholesterol (A) and triglycerides (B) in plasma from ApoE and ApoE/*mdx* mice as compared by the Mann-Whitney U was used.

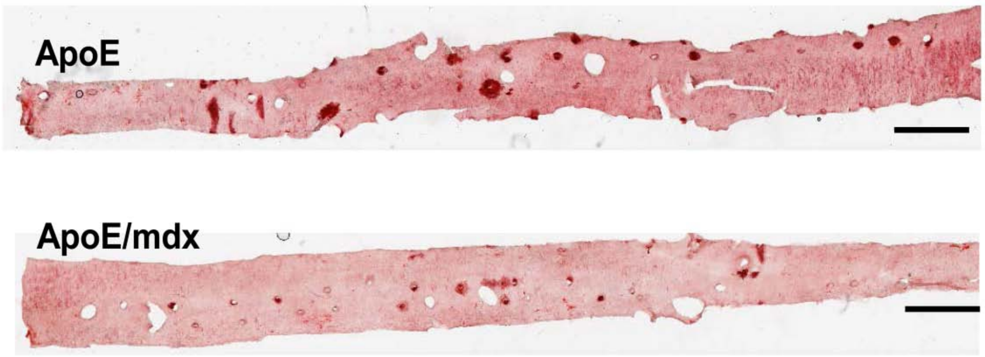

Supplementary figure S3: Aortic plaques. Flat preparations of aortas stained with Oil Red O from ApoE and ApoE/*mdx* mice. Scale bars represent 2 mm.

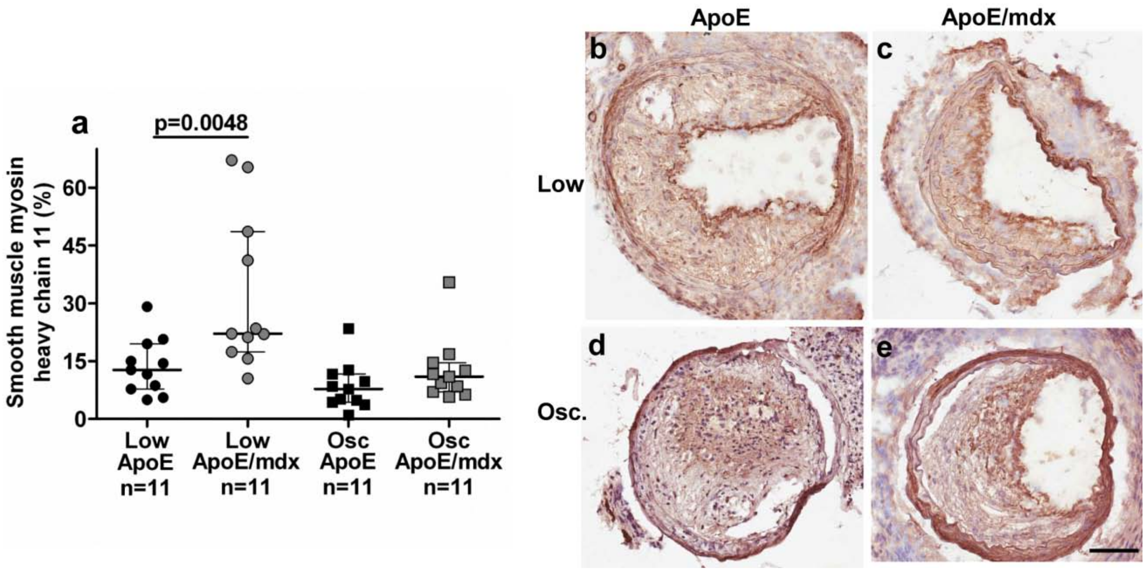

Supplementary figure S4: Plaque content of smooth muscle cells. Increased smooth muscle cell content as measured by smooth muscle myosin heavy chain in low shear stress plaques from ApoE/*mdx* mice compared to ApoE mice. Quantification is shown in a) and representative sections in b-e. Scale bars represent 100 µm. The statistical test used was the Mann-Whitney U test.

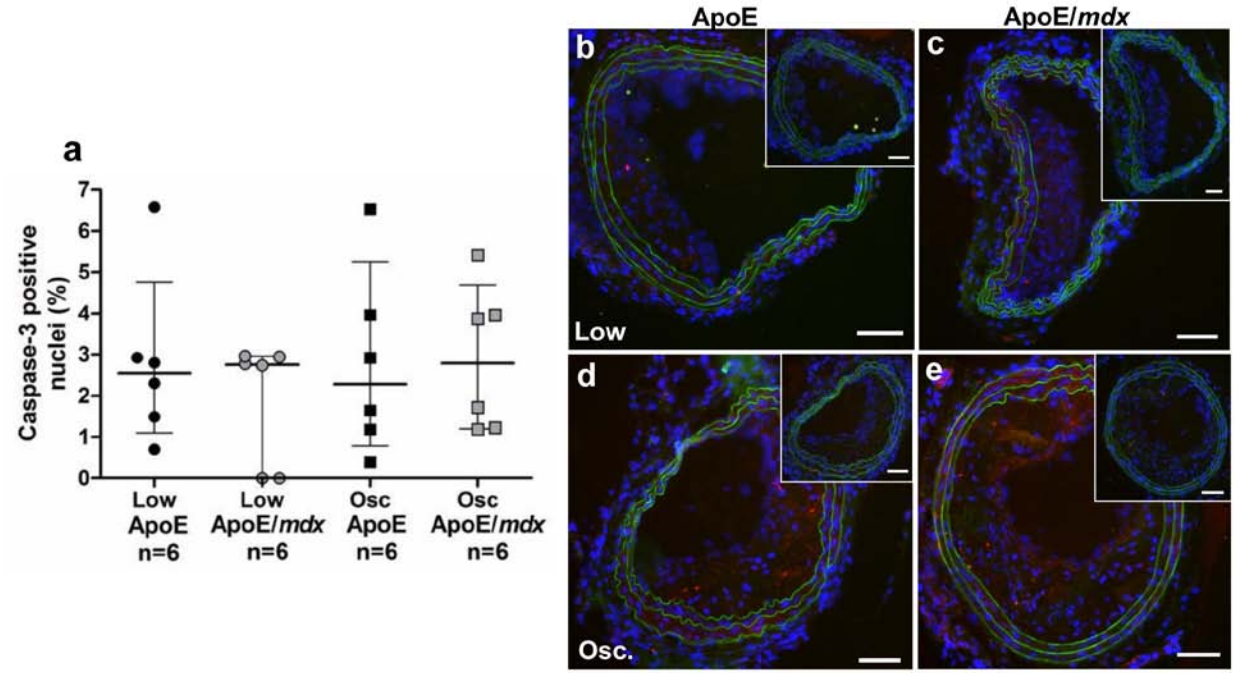

Supplementary figure S5: Apoptosis in carotid artery plaques. Quantification of caspase-3 positive cells as compared by the Mann Whitney U test (a) and representative images of caspase-3 immunofluorescence (red) in low and oscillatory shear stress plaques in ApoE and ApoE/mdx mice (b-e, with negative controls shown as insets). Nuclei are stained blue by DAPI and autofluorescence appears green. Scale bars are 100 µm.

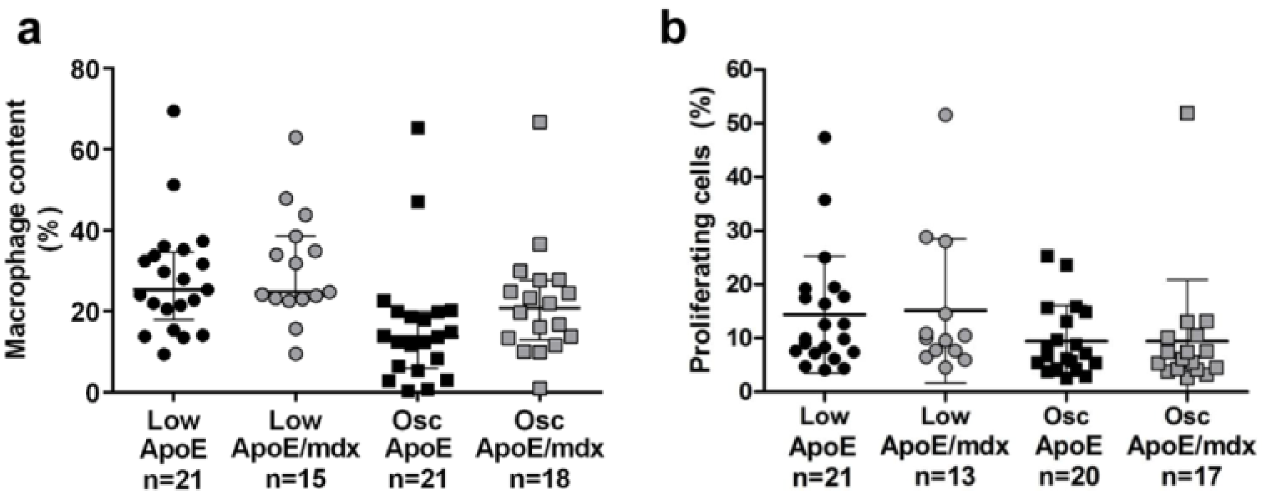

Supplementary figure S6. Plaque content of macrophages and proliferating cells. Content of macrophages (a; immunohistochemical marker mac2) and proliferating cells (b; immunohistochemical marker Ki67) normalised to plaque size was similar in low and oscillatory shear stress plaques from ApoE and ApoE/*mdx* mice as compared by the Mann-Whitney U test.

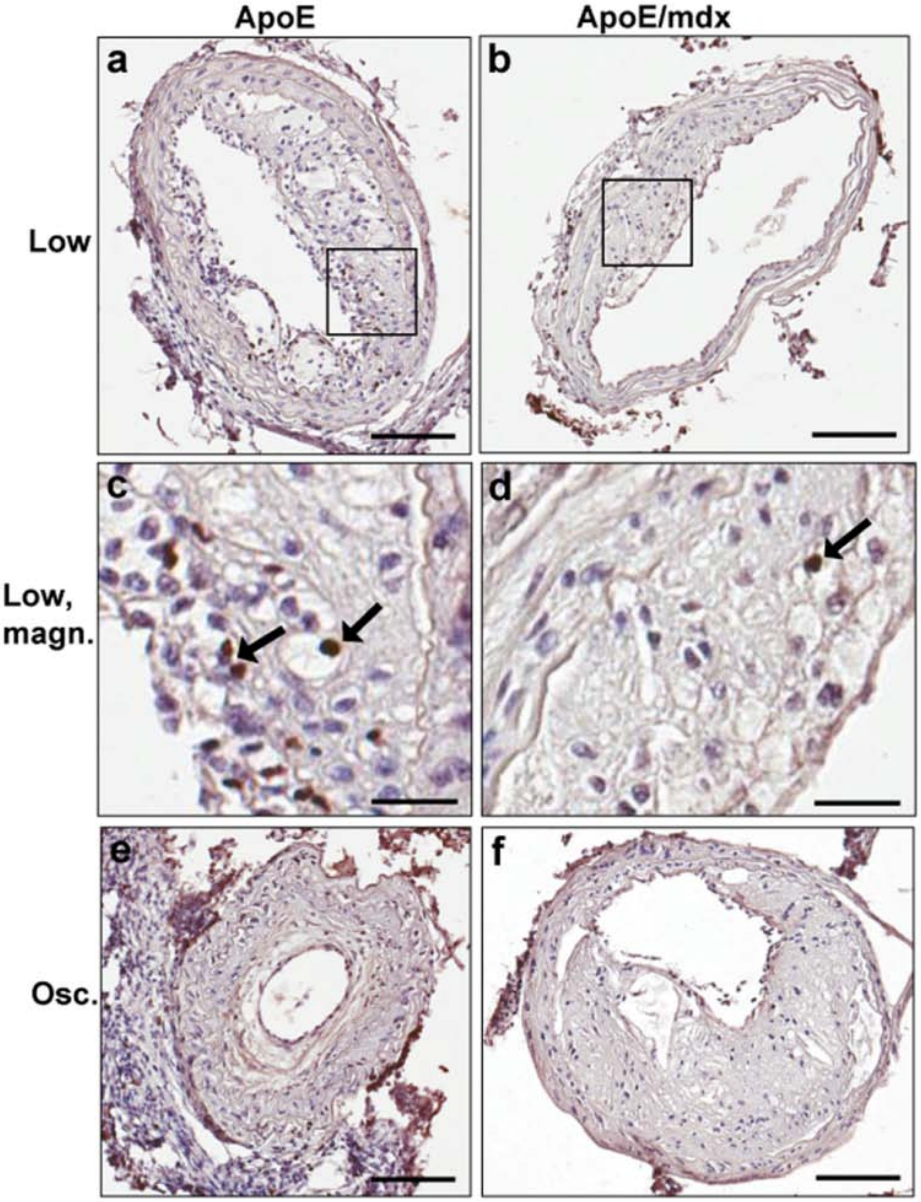

Supplementary figure S7: T cells in caroid artery plaques. Representative images showing CD3 immunoreactivity in low (a-b) and oscillatory (e-f) shear stress plaques from ApoE (a, c and e) and ApoE/*mdx* (b, d and f) mice with magnifications shown in c and d. Magnified areas are denoted by black squares. Scale bars in a-b and e-f represent 100 µm, and 25 µm in c-d.

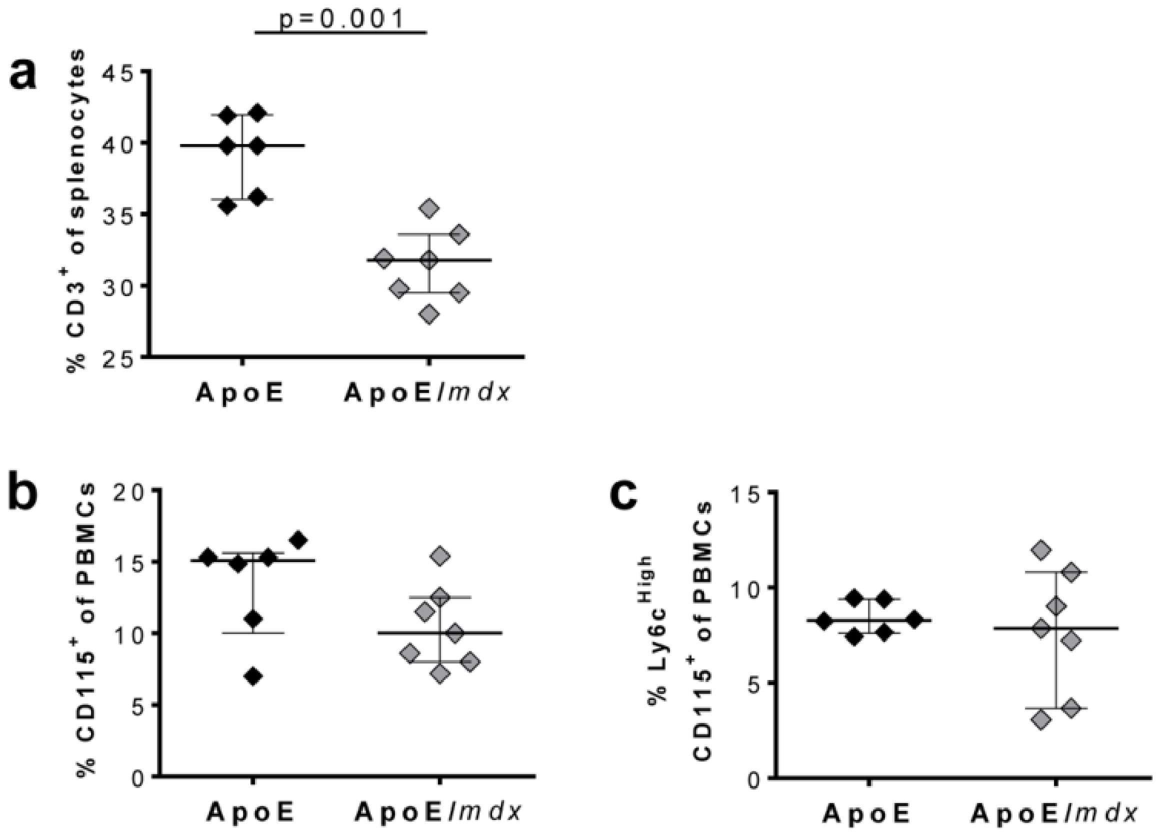

Supplementary figure S8: Lack of dystrophin in chow fed apoE mice results in decreased T cells. Quantification of frequencies of $CD3^+$ splenocytes (a), and $CD115^+$ (b) and $Ly6C^{High}$ $CD115^+$ (c) of PBMCs as compared by the Mann-Whitney U test.

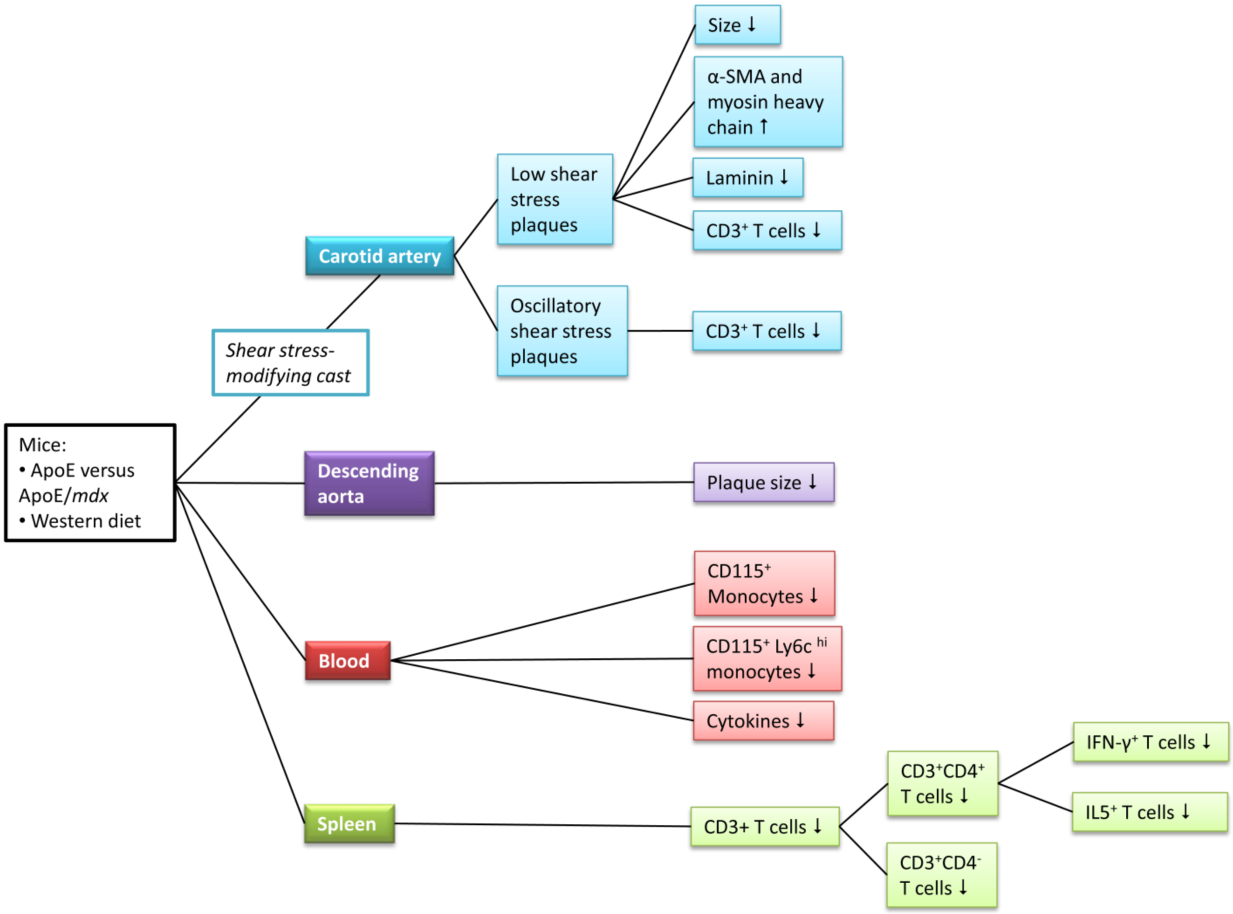

Supplementary figure S9: Overview of the results in the ApoE/*mdx* mouse. Flow chart summarizing the results found in ApoE/*mdx* mice grouped according to tissue type. Increased and decreased features are denoted by ↑ and ↓, respectively, and always refers to observed phenotypes in ApoE/*mdx* mouse tissue (in comparison with ApoE mouse tissue).

Supplementary table

Table S1. Cytokine levels in plasma from ApoE and ApoE/*mdx* mice on a chow diet (n=6 and 7, respectively). The Mann-Whitney U test was used and values are shown as median with interquartile range.

Cytokines	Plasma levels (pg/ml)		P values
	ApoE	ApoE/*mdx*	
IL-2	37.9 (17.0-85.5)	8.0 (7.2-8.8)	0.0023
IL-4	26.5 (10.5-39.9)	9.4 (7.2-12.5)	0.0221
IL-5	20.6 (9.4-43.1)	17.8 (16.8-20.8)	NS
IL-6	50.9 (34.7-76.1)	29.7 (23.6-31.2)	0.0140
IL-10	83.0 (32.8-168.3)	32.1 (26.5-44.5)	NS
IL-12p70	603.5 (307.6-934.9)	320.4 (295.9-431.7)	NS
IL-13	224.7 (111.1-463.1)	72.0 (65.2-97.6)	0.0023
IL-17	37.4 (22.1-76.4)	20.2 (10.2-27.3)	0.0480
IFNγ	10.2 (5.9-42.39)	5.9 (4.4-7.1)	NS
TNFα	886.6 (714.3-1027)	591.3 (465.3-885.2)	0.0350

Supplementary references

1. Cheng, C., *et al.* Atherosclerotic lesion size and vulnerability are determined by patterns of fluid shear stress. *Circulation* **113**, 2744-2753 (2006).
